# Treatment impact of newly approved therapeutic agents for metastatic urothelial carcinoma in Japan: a single-center retrospective study

**DOI:** 10.1038/s41598-023-43901-5

**Published:** 2023-10-03

**Authors:** Yuta Inoue, Takeshi Yamada, Atsuko Fujihara, Masatsugu Miyashita, Takumi Shiraishi, Masayoshi Okumi, Fumiya Hongo, Osamu Ukimura

**Affiliations:** Department of Urology, Kyoto Prefectural University of Medicine, Kamigyo-ku, Kyoto, 602-8566 Japan

**Keywords:** Bladder cancer, Cancer therapy, Chemotherapy

## Abstract

Although recent clinical trials of new therapeutic agents for metastatic urothelial carcinoma have shown prolonged overall survival, there are few real-world evidence. To assess the impact of new therapeutic agents, we performed retrospective analysis for consecutive 158 metastatic urothelial carcinoma patients who performed systemic therapy in our institution between May 2008 and August 2023. We defined a period from May 2008 to December 2017, when pembrolizumab was first introduced to the clinical setting in the new therapeutic agents for metastatic urothelial carcinoma in Japan, as “pre new drug era” and a period from January 2018 to August 2023 as “post new drug era”. We compared overall survival between pre- and post- new drug era using Kaplan–Meier method with log rank test. Median overall survival of pre- and post- new drug era were 14.5 months (95% confidence intervals: 11.6–16.7) and 23.1 months (95% confidence intervals: 14.5-NA), respectively (p < 0.001). Five-year survival rate of pre- and post- new drug era was 7.0% (95% confidence intervals: 2.3–15.3) and 36.3% (95% confidence intervals: 21.4–51.5), respectively. Multivariable Cox proportional hazards regression analysis of factors associated with overall survival showed that enfortumab vedotin administration, administration of second-line or more systemic therapy, best overall response of SD, PR and CR in first-line systemic therapy, higher serum albumin and lower CRP were factors for overall survival prolongation. Introduction of new therapeutic agents for metastatic urothelial carcinoma contributed to the improvement of overall survival in comparison with the era without these agents.

## Introduction

For metastatic urothelial carcinoma (mUC), pembrolizumab (Pem) prolonged median overall survival (mOS) 3 months compared to chemotherapy (10.3 months vs 7.4 months, p = 0.002) as a second-line treatment^1^. After the launch of Pem, Powles et al.^2^ reported in 2020 that avelumab (Ave) plus best supportive care prolonged mOS about 7 months compared to best supportive care alone as a maintenance therapy after the first-line chemotherapy (21.4 months vs 14.3 months, p = 0.001). Besides, Enfortumab vedotin (EV) prolonged mOS about 4 months compared to investigator-chosen chemotherapy after the refractory of platinum containing chemotherapy and immune-checkpoint inhibitor (ICI) (12.88 months vs 8.97 months, p = 0.001)^3^. These new drugs are currently covered by insurance in Japan (Pem can be used only after a second-line treatment in Japan). While combination of these new drugs would prolong the prognosis of mUC patients further, there are few reports to elucidate the influence of these new drugs toward the OS in the real-world.

We retrospectively investigated the effect of these new drugs in comparison between the era with and that without these drugs.

## Materials and methods

**Figure 1:**
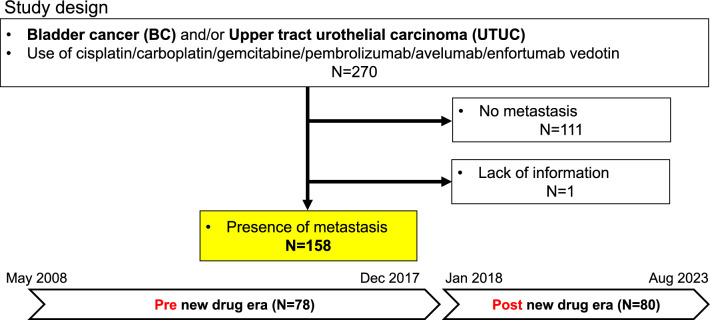
Flowchart of the patients’ inclusion and study design.

We retrospectively reviewed consecutive mUC patients who were performed systemic therapy between May 2008 and August 2023. We identified 270 bladder cancer (BC) and/or upper tract urothelial carcinoma (UTUC) patients who received cisplatin, carboplatin, gemcitabine, Pem, Ave and/or EV. We excluded 111 patients with no metastasis and 1 patient with insufficient information, and ended up to analyze 158 patients (Fig. 1).

We defined Pem, Ave and EV as “new drugs”. Since Pem was first launched in Japan and introduced in our institution at January 2018 for mUC, we defined a period from May 2008 to December 2017 as pre new drug era (pre-NDE) and a period from January 2018 to August 2023 as post new drug era (post-NDE). We defined pre-NDE as the last follow up date before December 2017, and post-NDE after January 2018. We compared OS between pre- and post-NDE using Kaplan–Meier method with log rank test.

The follow-up duration was defined from initial diagnosis of metastasis to last follow-up or death. Regional lymph node (LN) swelling was regarded as metastasis in patients who had received radical surgery, and was not included in metastasis if patients did not receive radical surgery. Urinary tract recurrence was not regarded as a metastasis. Ave was regarded as an independent regimen in order to facilitate evaluation of the treatment effect of Ave itself. Pem after neoadjuvant chemotherapy (NAC) and/or adjuvant chemotherapy (AC) was regarded as first-line even if recurrence was occurred within 1 year after NAC/AC. MVAC (methotrexate, vinblastine, adriamycin and cisplatin), GC (gemcitabine and cisplatin) and GCarbo (gemcitabine and carboplatin) were considered as a standard chemotherapy regimen. Each systemic therapy was determined according to the physician’s choice. All patients were followed at least every 3 months with routine blood tests and imaging tests including computed tomography, magnetic resonance imaging and/or positron emission tomography. The response of treatment was evaluated at each physician’s discretion.

This study was approved by the Research Ethics Committee of Kyoto Prefectural University of Medicine (ERB-C-1180-2) and conformed to the provisions of the Declaration of Helsinki. Written informed consent was obtained from all patients.

We found out each patient’s age, sex, performance status (PS), body mass index (BMI), charlson comorbidity index (CCI) and some blood test results such as serum albumin, C-reactive protein (CRP), estimated glomerular filtration rate (eGFR) and hemoglobin (Hb) using medical charts. Metastatic sites at the initial diagnosis of mUC were also checked, and classified into regional LN, distant LN, lung, bone, liver and others. Overall survival was calculated from the date of detecting initial metastasis to death or last follow-up. Toxicity that caused treatment discontinuation was described according to CTCAE v5.0.

All analyses were conducted using R version 4.1.2 and EZR^4^. Ordinal variable and nominal variable were statistically analyzed using Fisher’s exact probability test. Continuous variable was statistically analyzed using Mann–Whitney U test. Overall survival was estimated using the Kaplan–Meier survival curves with log-rank test. The multivariable Cox proportional hazard regression model was used to identify the prognostic factors for OS. Outcome measures were calculated with 95% confidence intervals (CI), and p-values < 0.05 were regarded significant.

We also performed inverse probability of treatment weighting (IPTW) method to adjust background for analyzing OS between groups. Propensity score was calculated by analyzing: age, sex, PS, BMI, CCI, serum albumin, CRP, eGFR, Hb, BC or UTUC, each metastatic site, cisplatin use, neutrophil–lymphocyte ratio (NLR), presence of perioperative chemotherapy, variant histology, the line of chemotherapy, presence of radical surgery and the number of initial metastatic organ.

### Ethical approval

This study was approved by the Research Ethics Committee of Kyoto Prefectural University of Medicine (ERB-C-1180-2) and conformed to the provisions of the Declaration of Helsinki. Written informed consent was obtained from all patients.

## Results

Seventy-eight patients were classified into pre-NDE and 80 patients into post-NDE. The details of patients’ characteristics were described in Table 1. There was significant difference in age (median 69.5 years old, range 38–88 for pre-NDE and 75.5 years old, range 48–87 for post-NDE, p < 0.001), serum albumin (median 4.0 g/dL, range 2.2–5.1 for pre-NDE and 3.8 g/dL, range 2.2–4.7 for post-NDE, p < 0.01) and eGFR (median 53.7 mL/min/1.73m^2^, range 20.9–135.2 for pre-NDE and 48.9 mL/min/1.73m^2^, range 5.43–111.3 for post-NDE, p < 0.05) between pre- and post-NDE. There was no significant difference between the groups with respect to sex, PS, BMI, CCI, CRP and Hb.

**Table 1:** Baseline characteristics of the patients at an initial diagnosis of metastasis.

		Pre new drug era	Post new drug era	p
		(N = 78)	(N = 80)	
Median age, years (range)		69.5 (38–88)	75.5 (48–87)	< 0.001
Median follow up period, months (range)		12.7 (1.18–85.5)	14.5 (0–127)	0.25
Sex, N	Male	61	57	0.36
	Female	17	23	
Median BMI, kg/m^2^ (range)		21.3 (14.9–28.7)	22.8 (14.9–32.8)	0.24
PS, N	0	28	28	0.69
	1	35	34	
	2	14	14	
	3	1	4	
CCI, N	0	33	38	0.63
	1	28	22	
	2	14	13	
	3	2	5	
	4	1	2	
Median serum albumin, g/dL (range)		4.0 (2.2–5.1)	3.8 (2.2–4.7)	< 0.01
Median CRP, mg/dL (range)		0.49 (0.01–13.9)	0.56 (0.02–15.9)	0.34
Median eGFR, mL/min/1.73m^2^ (range)		53.7 (20.9–135.2)	48.9 (5.43–111.3)	< 0.05
Median Hb, g/dL (range)		11.9 (7.6–16.6)	12.2 (7.6–15.5)	0.52
Tumor location, N	BC	34	38	0.64
	UTUC	44	42	
Presence of variant histology or non-UC, N		19	20	1.00
Cisplatin administration, N		52	53	1.00
History of radical surgery, N		46	36	0.08
Initial metastatic site, N	Regional lymph node	38	49	0.15
	Distant lymph node	22	23	1.00
	Visceral	42	34	0.20
	Liver	9	9	1.00
	Lung	22	14	0.13
	Bone	18	15	0.56
	Others	16	23	0.27
Number of initial metastatic site, N	1	45	42	0.58
	2	23	27	
	3	7	5	
	> = 4	3	6	

BMI, body mass index; PS, performance status; CCI, charlson comorbidity index; Alb, serum albumin; CRP, C-reactive protein; eGFR, estimated glomerular filtration rate; Hb, hemoglobin; mUC, metastatic urothelial carcinoma; BC, bladder cancer; UTUC, upper tract urothelial carcinoma; non-UC, non-urothelial carcinoma.

Tumor characteristics were also listed in Table 1. Eighty-six patients were UTUC (54.4%) and 39 patients had a variant histology or non-urothelial carcinoma (UC) histology (24.7%). The number of patients with the administration of cisplatin was 105 (66.5%) and of patients who received radical surgery was 82 (51.9%). There was no significant difference between the groups with respect to tumor location (BC or UTUC), presence of variant histology, cisplatin administration, history of radical surgery, initial metastatic site and the number of initial metastatic organ.

A total of 81 patients received new drugs (Pem 54, Ave 16 and EV 11). Eight patients received two new drugs. Median treatment duration of Pem, Ave and EV were 87 days (range: 1–1301 days), 99 days (range: 0–665 days) and 179 days (range: 91–611), respectively. Disease control rate (DCR) (stable disease (SD) + partial response (PR) + complete response (CR)) of Pem, Ave and EV were 35.2%, 56.3% and 90.9%, respectively (p = 0.0017) (Table 2).

**Table 2:** The outcome of new drugs.

	Total N	Median treatment duration, days (range)	BOR	first-line	second-line	third-line	fourth-line	Total	
Pem	54	87 (1–1301)	CR	0	1	0	0	1	19 (35.2%)
			PR	2	6	0	0	8	
			SD	2	7	0	1	10	
			PD	6	19	1	3	29	29 (53.7%)
			Unknown	1	3	1	1	6	6 (11.1%)
Ave	16	99 (0–665)	CR	0	0	0	0	0	9 (56.3%)
			PR	0	5	0	0	5	
			SD	0	4	0	0	4	
			PD	0	2	1	0	3	3 (18.7%)
			Unknown	0	4	0	0	4	4 (25.0%)
EV	11	179 (91–611)	CR	0	0	0	0	0	10 (90.9%)
			PR	0	1	7	0	8	
			SD	0	0	1	1	2	
			PD	0	0	1	0	1	1 (9.1%)
			Unknown	0	0	0	0	0	0 (0%)
			Total	11	52	12	6	81	
							p value	0.0017	

Pem, pembrolizumab; Ave, avelumab; EV, enfortumab vedotin; BOR, best overall response; CR, complete response; PR, partial response; SD, stable disease; PD, progressive disease. p value was analyzed using Fisher’s exact probability test.

**Figure 2:**
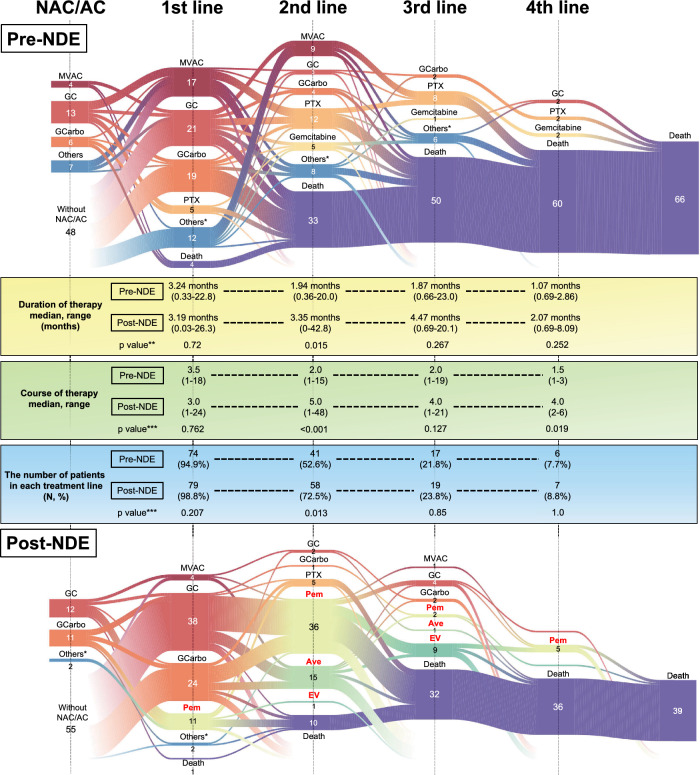
Sankey diagram which represents the detail of systemic therapy. Details of systemic therapy in pre-NDE and post-NDE are represented in upper and lower part of the figure, respectively. Duration of therapy in each treatment line is shown in middle yellow frame. Course of therapy in each treatment line is shown in middle green frame. The number of patients in each treatment line is shown in middle blue frame. NAC, neoadjuvant chemotherapy; AC, adjuvant chemotherapy; MVAC, methotrexate, vinblastine, adriamycin and cisplatin; GC, gemcitabine and cisplatin; GCarbo, gemcitabine and carboplatin; PTX, paclitaxel containing regimen; Pem, pembrolizumab; Ave, avelumab; EV, enfortumab vedotin. *Others: including GN (gemcitabine and nedaplatin), MVAN (methotrexate, vinblastine, Adriamycin and nedaplatin), EP (etoposide and cisplatin), ECarbo (etoposide and carboplatin), MEC (mitoxantrone, etoposide and cytarabine), intra-arterial chemotherapy, Nivolumab (clinical trial) and tegafur-uracil. **p value was analyzed using Mann–Whitney U test, ***p value was analyzed using Fisher’s exact probability test.

Details of systemic therapy was described by Sankey diagram (Fig. 2). Sixty-six patients in pre-NDE and thirty-nine patients in post-NDE died during the follow-up period. Paclitaxel containing regimen, gemcitabine monotherapy or other regimens were generally used after the standard regimen (MVAC, GC and GCarbo) in pre-NDE. In the second-line, patients in post-NDE received significantly longer duration and more courses of therapy than those in pre-NDE. Besides, significantly greater rate of patients received second-line systemic therapy in post-NDE than that in pre-NDE (72.5% and 52.6%, respectively. p = 0.013). Table 3 shows DCR of each systemic therapy line. In the third-line, DCR was significantly better in post-NDE (13/16, 76.5%) than in pre-NDE (6/17, 35.3%) (p = 0.037).

**Table 3:** Best overall response of each line of systemic therapy.

		Pre new drug era (N = 78)	Post new drug era (N = 72)	Overall (N = 150)	p
1st line	SD + PR + CR	43	50	93	0.61
	PD	29	27	56	
2nd line	SD + PR + CR	14	27	41	0.34
	PD	26	30	56	
3rd line	SD + PR + CR	6	13	19	0.037
	PD	11	4	15	
4th line	SD + PR + CR	1	3	4	0.55
	PD	5	3	8	

SD, stable disease; PR, partial response; CR, complete response; PD, progressive disease.

A total of 14 patients discontinued drugs because of the toxicity (3 patients in first-line, 8 in second-line and 3 in third-line). Among them, 4 patients discontinued chemotherapy (1 GCarbo: grade 4 thrombocytopenia; 3 GC: grade 3 hyponatremia, grade 4 neutropenia and grade 4 thrombocytopenia), 8 discontinued ICI (5 Pem: grade 3 liver dysfunction, grade 2 hyponatremia, grade 3 diarrhea, grade 2 uveitis and grade 2 encephalitis; 3 Ave: grade 2 infusion reaction, grade 2 adrenal failure and grade 2 arthritis) and 2 discontinued EV (grade 2 peripheral neuropathy and grade 3 interstitial pneumoniae). Eight patients proceeded to the next-line systemic therapy. No patients died due to adverse events.

**Figure 3:**
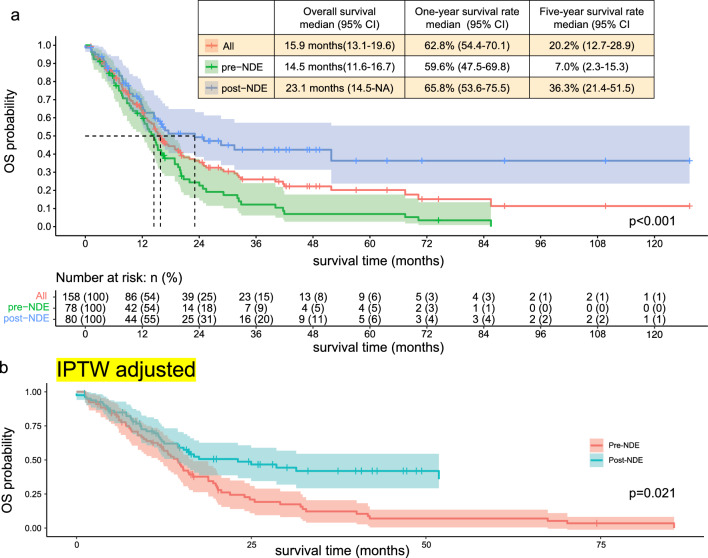
(**a**) Kaplan-Meyer curve representing overall survival of the whole patients, pre-NDE patients and post-NDE patients. The red line represents overall survival curve of whole patients. The green line with ribbon represents overall survival curve with 95% confidence interval of pre-NDE patients. The blue line with ribbon represents overall survival with 95% confidenve interval of post-NDE patients. pre-NDE, pre new drug era; post-NDE, post new drug era. (**b**) Kaplan-Meyer curve of overall survival between pre-NDE and post-NDE after IPTW adjusting. The red and green line with ribbon represents overall survival with 95% confidence interval of pre-NDE and post-NDE, respectively. pre-NDE, pre new drug era; post-NDE, post new drug era; IPTW, inverse probability of treatment weighting.

Overall survival of the whole 158 patients was estimated by the Kaplan–Meier survival curves (Fig. 3a). Median OS was 15.9 months (95% CI: 13.1–19.6 months). One- and five-year survival rate were 62.8% (95% CI: 54.4–70.1) and 20.2% (95% CI: 12.7–28.9), respectively. Next, we compared OS between pre- and post- NDE (Fig. 3a). Median OS of pre- and post-NDE was 14.5 months (95% CI: 11.6–16.7) and 23.1 months (95% CI: 14.5-NA), respectively (p < 0.001). One-year and five-year survival rate of pre-NDE were 59.6% (95% CI: 47.5–69.8) and 7.0% (95% CI: 2.3–15.3), respectively. One-year and five-year survival rate of post-NDE were 65.8% (95% CI: 53.6–75.5) and 36.3% (95% CI: 21.4–51.5), respectively. We also used IPTW method to reduce the effects of confounders (Fig. 3b). Even after IPTW adjustment, there were statistically significant OS prolongation in post-NDE compared to pre-NDE (p = 0.021).

**Figure 4:**
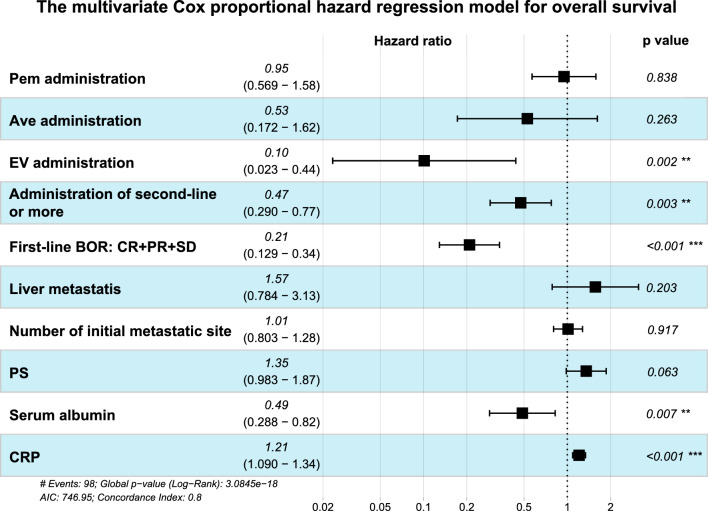
The multivariable Cox proportional hazard regression model to identify the prognostic factors for overall survival. A forest plot of the analysis are listed, and the dashed line indicates a hazard ratio of 1. Pem, pembrolizumab; Ave, avelumab; EV, enfortumab vedotin; BOR, best overall response; CR, complete response; PR, partial response; SD, stable disease; PS, performance status; CRP, C-reactive protein.

Next, we performed multivariable Cox proportional hazard model analyses to identify factors associated with OS. EV administration (hazard ratio 0.10, 95% CI: 0.23–0.44, p = 0.002), administration of second-line systemic therapy or more (hazard ratio 0.47, 95% CI: 0.29–0.77, p = 0.003), first-line best overall response (BOR) of CR, PR and SD (hazard ratio 0.21, 95% CI: 0.13–0.34, p < 0.001), higher serum albumin (hazard ratio 0.49, 95% CI: 0.29–0.82, p = 0.007) and lower CRP (hazard ratio 1.21, 95% CI: 1.09–1.34, p < 0.001) were the significant favorable prognostic factor for OS (Fig. 4).

## Discussion

In the present study, we elucidated the significant OS improvement in the post-NDE compared to pre-NDE (median 23.1 months vs. 14.5 months, respectively) in the real-world data in Japan before and after IPTW adjustment. Although new drugs for mUC achieved significant OS prolongation compared to placebo or chemotherapy in the clinical trials^1–3^, there were few previous reports to assess the effect of new drugs in the real-world data.

Narita et al.^5^ reported the significant difference in the mOS between Pem group and chemotherapy group by the IPTW method (24.7 months vs. 16.3 months, p = 0.159 in the raw data and p = 0.003 in the IPTW method) in the advanced UC patients. Taguchi et al.^6^ reported that Pem use prolonged mOS compared to control group (24 months vs. 11 months, p < 0.0001) in advanced UC patients. Taken together, mOS of advanced UC patients who received at least second-line Pem was 24 months in the real-world. These reports and our study were the data from Japan, and new drugs (Pem, Ave and EV) were used according to the Japanese healthcare system. The issues are that these previous reports included only patients who received at least second-line systemic therapy and did not analyze patients who received only first-line systemic therapy.

In the present study, administration of second-line systemic therapy or more was one of the prognostic factors (Fig. 4). Aly et al.^7^ reported mOS of each line of chemotherapy. Median OS of patients who received second-line (16.6 months, 95% CI: 15.2–19.2) and third-line (29.8 months, 95% CI: 26.2–33.5) chemotherapy was longer than mOS of patients received only first-line chemotherapy (12.4 months, 95% CI: 11.7–13.4). In this study, significantly more patients received second-line in post-NDE than those in pre-NDE. Besides, patients in post-NDE received significantly longer duration and greater courses of second-line than patients in pre-NDE (Fig. 2), which might be owed to the launch of new drugs. In addition, there was no significant difference in the duration and courses of first-line between pre- and post-NDE. It implies that the situation of first-line systemic therapy has not changed even though the introduction of new drugs.

Multivariable Cox proportional hazard model analyses revealed that lower serum albumin and higher CRP were significant adverse prognostic factors (Fig. 4). Hypoalbuminemia was reported to be a biomarker of systemic inflammation and malnutrition, and a prognostic marker in some cancers^8,9^. There were several reports about preoperative hypoalbuminemia in non-metastatic UC patients (^10,11^, whereas there were few reports of hypoalbuminemia in mUC patients. Park JH analyzed mUC patients receiving ICI after platinum-containing chemotherapy and reported that hypoalbuminemia, NLR > 5 and liver metastasis were the independent prognostic factor for adverse OS^12^. High CRP level indicated cancer-related inflammation^13^ and significantly associated with worse OS in mUC patients^14,15^. Though there were a variety of other prognostic factors for mUC patients which were previously reported (e.g., PS, visceral metastasis, liver metastasis, NLR, Hb, etc.)^10–16^, PS and liver metastasis were not prognostic factors in our study. We described PS according to the medical record, which might be less accurate than prospectively recorded information. Patients with liver metastasis in our study (18 patients, 11.4%, Table 1) were fewer than patients in previous reports (20.5–29%)^12,16^, which might be a factor of bias.

Best overall response of CR, PR and SD in first-line systemic therapy was significant favorable prognostic factor in our report (Fig. 4). Kato et al., analyzed 391 mUC patients who received first-line chemotherapy followed by second-line Pem. The patients who responded to first-line chemotherapy (PR or CR) had longer OS than the patients who did not respond^17^. Li et al.^18^ reported that poor first-line chemotherapy response was associated with higher risk of disease progression.

Kaplan-Meyer curve in this study showed that there was only 6.4% difference of 1-year survival rate between pre- and post-NDE (59.6% and 65.8%, respectively) and that two curves were close during the first 12 months, which also suggested that non-responders of the first-line systemic therapy had poor prognosis and that the effect of systemic therapy in a very-early phase was not improved in post-NDE (Fig. 3b).

We are able to use new drugs only after the standard chemotherapy in Japan at the time of this study. Some clinical trials which investigated ICI plus standard platinum containing chemotherapy did not show statistically significant OS prolongation compared to chemotherapy^19–21^. To achieve further improvement of OS, it is essential to find out new effective first-line systemic therapy for mUC.

We aim to reveal the influence of new drugs toward the OS in the real-world. Our result showed that among new drugs, EV administration was the only significant prognostic factor in the multivariable Cox proportional hazard model (Fig. 4).

Ave is performed as a maintenance therapy for patients who achieved durable response of first-line platinum chemotherapy, whereas Pem is often administered to patients who had progressive disease. In the current study, DCR of Pem and Ave were 35.2% and 56.3%, respectively. Li et al.^18^ reported that the poor response of first-line chemotherapy was associated with worth ICI treatment response, which might explain the worse DCR of Pem than that of Ave in our study.

Our report described that EV achieved DCR of 90.9% even after the administration of Pem and Ave. Previous reports supported this finding. In the EV-301 trial, DCR of EV was 71.9% (95% CI: 66.3–77.0)^3^. In the real world, Minato et al.^22^ recently reported that DCR of EV was 80.8%. Besides, previously reported DCR of Pem and Ave were 66.9% (CR: 2.5%, PR: 27.2% and SD: 37.2%)^5^ and 22.3% (CR: 6.0%, PR: 3.7% and SD: 12.6%)^2^, respectively. In summary, EV would achieve better disease control compared to Pem and Ave, which supported the result of the multivariable Cox proportional hazard model analysis.

There were some limitations in this report. Small sample size, single center analysis and retrospective nature of this study might be factors of bias. Although we used IPTW method, it might be difficult to reduce time bias of comparing pre-NDE and post-NDE.

The novelty of this study is using the first Japanese real-world data containing Ave and EV as well as Pem. This report was composed of non-adjusting raw data, which reflected the actual real-world situation in Japan. Notably, our study included wide-range of treatment selections (from patients with only first-line chemotherapy to patients with fourth-line systemic therapy), which could show mOS in the real-world setting compared to previous reports.

In conclusion, OS for mUC in the post-NDE improved significantly compared to pre-NDE. The significant prognostic factor to prolong OS in mUC patients included EV administration, second-line or more systemic therapy, SD or better response to the first-line systemic therapy, higher serum albumin and lower CRP. Especially, EV administration was a strong prognostic factor. In the current clinical practice, patients should receive more effective first-line chemotherapy, should be administered second-line Pem or maintenance Ave and should be performed EV in order to achieve better OS.

## Data Availability

The datasets used and/or analyzed in the present study available from the corresponding author on reasonable request.
